# Supplementary material for: Mechanisms governing the pioneering and redistribution capabilities of the non-classical pioneer PU.1
Source: Nat Commun. 2020 Jan 21;11:402. doi: 10.1038/s41467-019-13960-2 (PMC6972792; doi:10.1038/s41467-019-13960-2)
Supplement: Supplementary file 7 — Source data [file 41467_2019_13960_MOESM7_ESM.zip › Source_Data/Figure5/Figure5A_MotifScanOutput/homerResults/motif54.similar.html]

motif54

## Information for motif54

T
G
A
C
A
C
G
T
C
A
T
G
C
G
T
A
T
C
A
G
G
T
A
C
A
C
T
G
A
T
G
C
G
A
C
T
C
A
G
T
  
Reverse Opposite:  

G
T
C
A
C
T
G
A
T
A
C
G
G
T
A
C
C
A
T
G
A
G
T
C
G
C
A
T
G
T
A
C
T
G
C
A
A
C
T
G
  

|  |  |
| --- | --- |
| p-value: | 1e-29 |
| log p-value: | -6.697e+01 |
| Information Content per bp: | 1.628 |
| Number of Target Sequences with motif | 639.0 |
| Percentage of Target Sequences with motif | 21.19% |
| Number of Background Sequences with motif | 6115.1 |
| Percentage of Background Sequences with motif | 13.41% |
| Average Position of motif in Targets | 265.2 +/- 225.5bp |
| Average Position of motif in Background | 208.9 +/- 136.5bp |
| Strand Bias (log2 ratio + to - strand density) | -0.0 |
| Multiplicity (# of sites on avg that occur together) | 1.12 |
| Motif File: | file (matrix) reverse opposite |

### Similar de novo motifs found

|  |  |  |  |  |  |  |  |
| --- | --- | --- | --- | --- | --- | --- | --- |
| Rank | Match Score | Redundant Motif | P-value | log P-value | % of Targets | % of Background | Motif file |
